# Worldwide productivity and research trend of publications concerning tumor immune microenvironment (TIME): a bibliometric study

**DOI:** 10.1186/s40001-023-01195-3

**Published:** 2023-07-10

**Authors:** Yao-Ge Liu, Shi-Tao Jiang, Lei Zhang, Han Zheng, Ting Zhang, Jun-Wei Zhang, Hai-Tao Zhao, Xin-Ting Sang, Yi-Yao Xu, Xin Lu

**Affiliations:** Department of Liver Surgery, Peking Union Medical College Hospital, Chinese Academy of Medical Sciences & Peking Union Medical College (CAMS & PUMC), Beijing, China

**Keywords:** Tumor immune microenvironment (TIME), Bibliometrics, VOSviewer, Citespace, Frontiers

## Abstract

**Background:**

As the complexity and diversity of the tumor immune microenvironment (TIME) are becoming better understood, burgeoning research has progressed in this field. However, there is a scarcity of literature specifically focused on the bibliometric analysis of this topic. This study sought to investigate the development pattern of TIME-related research from 2006 to September 14, 2022, from a bibliometric perspective.

**Methods:**

We acquired both articles and reviews related to TIME from the Web of Science Core Collection (WoSCC) (retrieved on September 14, 2022). R package “Bibliometrix” was used to calculate the basic bibliometric features, present the collaborative conditions of countries and authors, and generate a three-field plot to show the relationships among authors, affiliations, and keywords. VOSviewer was utilized for co-authorship analysis of country and institution and keyword co-occurrence analysis. CiteSpace was used for citation burst analysis of keywords and cited references. In addition, Microsoft Office Excel 2019 was used to develop an exponential model to fit the cumulative publication numbers.

**Results:**

A total of 2545 publications on TIME were included, and the annual publication trend exhibited a significant increase over time. China and Fudan University were the most productive country and institution, with the highest number of publications of 1495 and 396, respectively. Frontiers in Oncology held the highest number of publications. A number of authors were recognized as the main contributors in this field. The clustering analysis revealed six clusters of keywords that highlighted the research hot spots in the fields of basic medical research, immunotherapy, and various cancer types separately.

**Conclusions:**

This research analyzed 16 years of TIME-related research and sketched out a basic knowledge framework that includes publications, countries, journals, authors, institutions, and keywords. The finding revealed that the current research hot spots of the TIME domain lie in “TIME and cancer prognosis”, “cancer immunotherapy”, and “immune checkpoint”. Our researchers identified the following areas: “immune checkpoint-based immunotherapy”, “precise immunotherapy” and “immunocyte pattern”, which may emerge as frontiers and focal points in the upcoming years, offering valuable avenues for further exploration.

**Supplementary Information:**

The online version contains supplementary material available at 10.1186/s40001-023-01195-3.

## Introduction

In recent years, with the widespread application of immunotherapy in multiple cancers, the role of tumor immune microenvironment (TIME) is getting the increasing attention it deserves. The tumor immune microenvironment derives from the concept of the tumor microenvironment (TME), which is defined to be composed of heterogeneous cancer cells and multiple stromal cell types along with associating parenchyma [1]. However, TIME concentrates more on understanding the complexity and diversity of immunological components of TME.

The studies of TIME burst in recent years, although with limited research history. Currently, three main classes of TIME have been recognized to describe the immunological frequency and cellular status infiltrated-excluded (I-E) TIME, infiltrated–inflamed (I-I) TIME, and tertiary lymphoid structures (TLS) TIME [2]. (I-E) TIME is characterized by cytotoxic lymphocytes (CTLs) located on the border of the tumor but poorly infiltrated into the tumor core, which is hypothesized to be poorly immunogenic and characterized by immunological ignorance [3]. I-E TIME can be found in several epithelial cancers, such as colorectal carcinoma [4] and pancreatic ductal adenocarcinoma [5]. Leukocytes and tumor cells that express the PD-1 (programmed cell death protein 1) ligand PD-L1 (programmed death-ligand 1) and CTLs that express PD-1 are more prevalent in I-I TIME, which dampens the immune response. Microsatellite instability high (MSI-H) colorectal cancer is one representative of this TIME type which has higher responses to immune-checkpoint blockade treatment [6]. TLS-TIME is a subclass of I-I TIME containing TLSs, including T cells, B cells, and Treg cells which is often associated with a positive prognosis for cancer patients. However, the characteristic of TLS requires further elucidation [7]. Furthermore, studies unveil the inextricable relationship between tumor cells and TIME. Tumor genotypes and phenotypes contribute to the formation of TIME through processes such as cytokine production [8] and stroma modulation [9]. Despite deeper understanding and explosive expansion of published articles in the field of TIME, there is still a dearth of precise and useful information concerning the publication status of relevant literature in this field.

The bibliometric analysis serves as a valuable tool for delving into the developmental trajectory, current research trends, and promising avenues of investigation within a specific research domain. By harnessing various indicators such as references, authors, journals, countries, and institutions, this approach enables quantitative analysis of an extensive body of scholarly literature [10]. In contrast to traditional systematic reviews and meta-analyses, bibliometric analysis can provide a more systematic and intuitive perspective to uncover the evolutionary path of a research topic. However, the extensive feature of TIME, encompassing numerous cancer types, poses significant challenges for meta-analyses. Comprehensive analysis of the entire research field becomes impractical due to this diversity. This limitation arises from the heterogeneity of different tumors and the variations in evaluation criteria. Consequently, existing meta-analyses in the field of TIME are often constrained to analyzing specific tumor types individually [11, 12], making it challenging for readers to gain a comprehensive understanding of this domain. By presenting a wealth of data through knowledge maps, we used CiteSpace, VOSviewer, as well as Bibliometrix R package as the main software tools to analyze and paint descriptive images as well as collaboration network maps based on the Web of Science database [13, 14] to visually showcase essential information from relevant literature in the field of TIME.

In comparison to similar latest research and after reviewing the existing literature, we discovered that there is a scarcity of bibliometric research on TIME. In addition, the domains covered by these articles may be fragmented. Several bibliometric analyses have focused on the tumor microenvironment of specific tumors. Wu et al. [15] conducted a bibliometric analysis to reveal the research trends in the tumor environment of pancreatic cancer using CiteSpace and VOSviewer as tools. The research findings indicate that research hot spots primarily revolve around several key areas including energy metabolism, cancer-associated fibroblasts, accurate diagnosis, drug delivery, and new treatments. In a bibliometric analysis conducted by Chen et al. [16] on the literature on hematological tumors, research trends related to the tumor microenvironment were examined. The analysis revealed that the predominant research emphasis is on targeted immunotherapy. Apart from this, specific type of tumor microenvironment has also been studied using bibliometric analysis. Zhang et al. [17] presented a comprehensive review of research conducted in the field of inflammatory tumor microenvironment spanning several decades and revealed inflammation, immunity, and angiogenesis as research hot spots. More recently, in 2023, articles employing bibliometric analysis in the field of TIME are still being produced and made significant contributions by exploring various angles, including but not limited to delving into the immunological mechanisms underlying photothermal therapy to enhance its effectiveness [18] and offering valuable guidance and novel perspectives in the field of tumor-associated macrophages [19].

In the whole, existing bibliometric articles in the field of TIME have summarized and analyzed the current state of research from various perspectives, offering valuable insights and prospects for future research directions. However, the existing studies lack a comprehensive understanding of the overall landscape of TIME, and no bibliometric study on the topic of TIME has ever been published.

Based on an analysis of the current research status and an overview of the existing research hot spots, this study sought to investigate the development pattern of TIME-related research from 2006 to September 14, 2022, from a bibliometric perspective. More specifically, this research aims to achieve the following key objectives:

1. To describe the current research status, such as publication analysis from various aspects, including country, institution, authorship, and journals.
2. To perform co-occurrence networks analysis of keywords and discover emerging research trends in this area.
3. To assist researchers in identifying potential avenues for future research exploration in the field of TIME

## Methods

### Data source acquisition and search strategy

In our study, relevant literature was searched and extracted from the Web of Science Core Collection (WoSCC) database as the data source before September 14, 2022. Four indexes from the WoSCC, the Science Citation Index Expanded (SCI-Expanded), the Social Sciences Citation Index (SSCI), the Arts & Humanities Citation Index (A&HCI), and the Emerging Sources Citation Index (ESCI) were included in the study. We selected the Web of Science (WoS) database in this research based on several reasons. Just as it has been utilized in scholarly literature research, WoSCC is one of the most influential academic databases, containing more than 20,000 journals covering a wide range of academic disciplines, including natural sciences, engineering and technology, and medicine. WoS is a widely accepted and commonly utilized database for producing citation data analysis and various evaluation purposes [20] and has been used in numerous bibliometric analyses, serving as a fundamental resource for analyzing and assessing scholarly publications [21–24]. In addition, although other databases such as Scopus and PubMed have been applied to bibliometric study [25–27], they both have their own limitations. The PubMed database may introduce biases in the search approach that prioritize medical domains, while WoS provides a more powerful citation analysis feature (such as the “Cited Reference Search” function) than the Scopus database that allows researchers to track and assess the citation status of publications, enabling them to understand their impact and academic influence. Other limitations of the Scopus database include: it lacks references published prior to 1996 and research results in the Scopus database cannot be presented by institutions and countries like WoS [28], which were essential components of our analysis. Considering the factors mentioned above, we selected the WoSCC database for conducting further analysis.

The search strategy was set as follows: [TS = (“Tumor immune microenvironment” OR “Tumor immune microenvironments” OR “Tumour immune microenvironment” OR “Tumour immune microenvironments” OR “Cancer immune microenvironment” OR “Cancer immune microenvironments” OR “Immune microenvironment of tumor” OR “Immune microenvironment of tumors” OR “Immune microenvironment of tumour” OR “Immune microenvironment of tumours” OR “Immune microenvironment of cancer”)]. The document types included research articles and reviews, and the publication language was specified as English. All the publications were extracted and saved in plain.txt format (including full records and cited references) for further analysis.

### Data analysis

This bibliometric analysis was performed mainly using three tools R version 4.0.1 [29], VOSviewer (version 1.6.18) [30], and CiteSpace (version 5.2R) [31].

Bibliometrix version 4.0.0 is an R package involving functions for bibliometrics and scientometrics quantitative research. In this research, it was used to (1) present the number of publications and citations of bibliometric analysis; (2) determine the frequency of keywords and terms; (3) analyze the collaboration frequency among countries and authors; and (4) establish a three-field plot to visualize keyword analysis. Bibliometrix is an R package that can be utilized for bibliometric analysis using its default parameters.

VOSviewer is a bibliometric software concentrating on the bibliometric network visualization based on the distance of plots [32]. It can construct co-occurrence networks of specific terms and provide overlaying visual network maps over time, in which the color and distance of plots represent their distribution in time and association accordingly [33]. In this study, we primarily used VOSviewer to perform: (1) co-authorship analysis to explore the cooperative relationships among authors and their institutions and (2) co-occurrence analysis to explore the relations among the keywords.

The thresholds of VOSviewer were set as follows—co-authorship analysis: frequency of at least 7; keyword co-occurrence: frequency of at least 20; co-authorship analysis of institution: the top 100 most productive institutions. The remaining functionalities of VOSviewer could be analyzed using the default parameters.

CiteSpace is a citation visualization tool which can generate the knowledge maps of main information such as countries, authors, keywords, institutions and co-citations. We mainly used it in our study to find highly cited references and keywords that experienced citation bursts during a period of time, which indicates emerging research hot spots. The thresholds of VOSviewer were set as follows: look back years, 8; link retaining factor, 3; percentage of nodes to label, 1.0%; maximum links per node, 5; TopN = 2.0; filter refs by intrinsic citation, on.

In addition, an international collaboration map among countries was performed using the online website (https://bibliometric.com/). Microsoft Office Excel 2019 (Microsoft, Redmond, Washington, USA) was used to process the data and build an exponential model to fit the cumulative number of publications.

## Results

### Publication growth trend

**Fig. 1:**
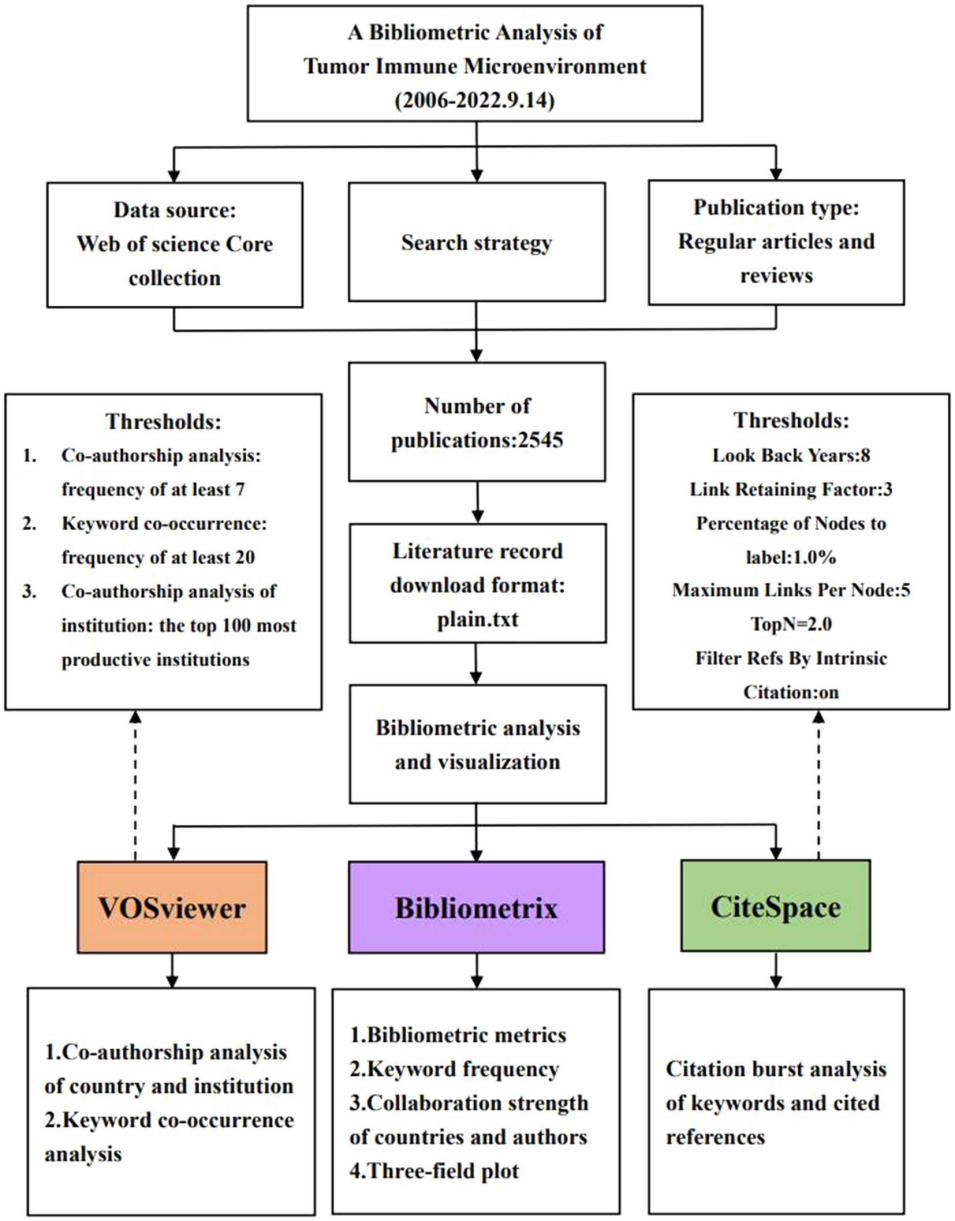
Diagram of the process and key steps of the study

**Fig. 2:**
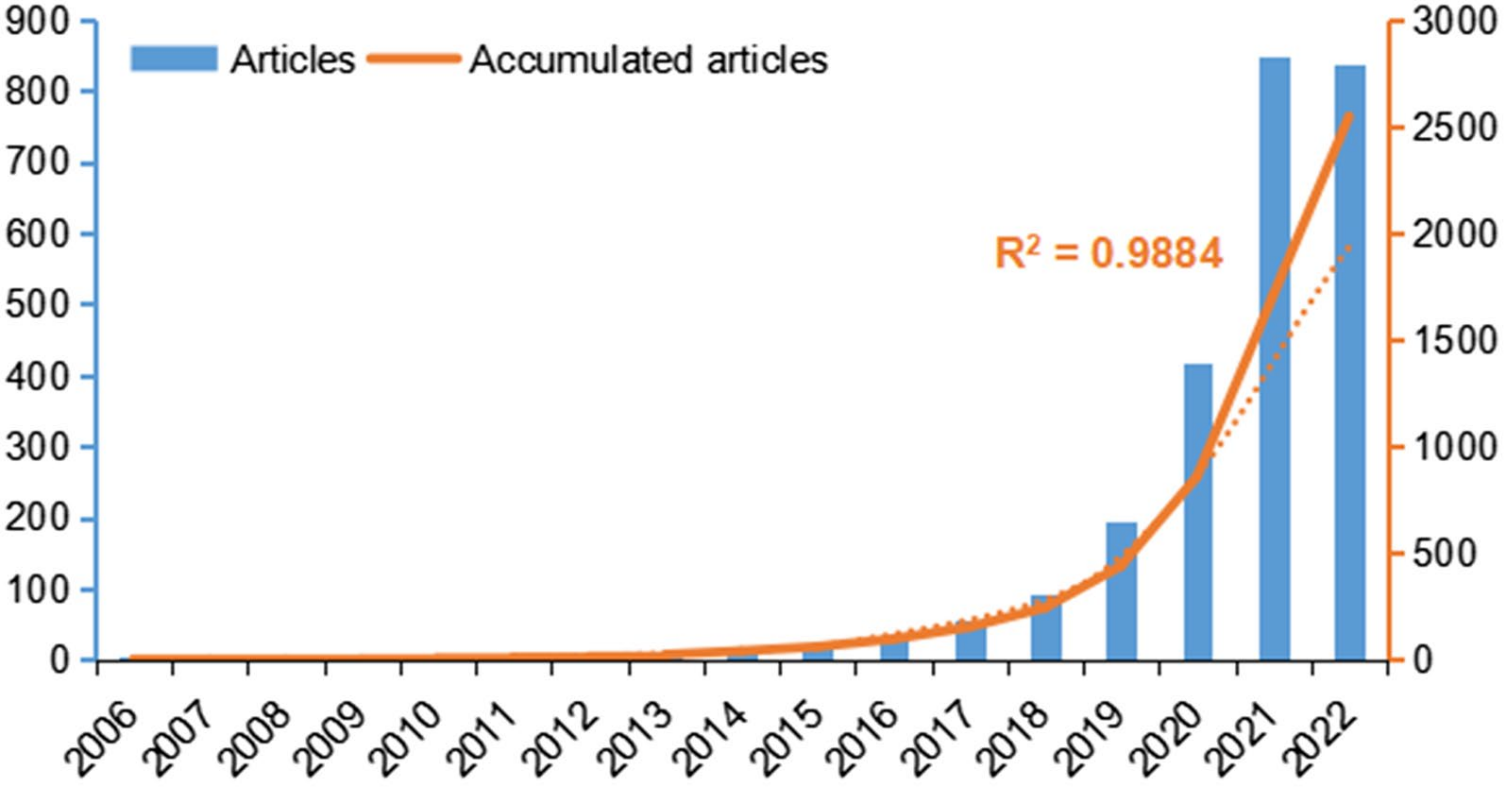
The annual publications between 2006 and 2022.9.14 and the cumulative articles

A flowchart to summarize this study is shown in Fig. 1, with thresholds used in the Vosviewer and Citespace labeled. A total of 2545 articles concerning TIME, which were published between 2006 and September 14, 2022, were obtained from the Web of Science database. As shown in Fig. 2, we can see the quantity and trend of annual publication numbers. The Blue column represents the annual publication numbers and the orange dotted curve represents the exponential model fitting the cumulative number of publications. Based on the search strategy we have employed, the earliest identified article dated back to 2006, and the annual number of publications remained less than 10 before 2014. In 2007 and 2008, there was no publication in this field. The annual publications have increased steadily since the year of 2018 and reached a peak in 2021 (*n* = 847). Until September 14, 2022, the publications concerning TIME in 2022 has reached 839, showing great potential to surpass the annual publication numbers in the past years. Additionally, we observed the significant effects of the exponential model fitting the cumulative number of publications per year (*R*^2^ = 0.9884) before 2022.

### Analysis of countries and institutions

**Fig. 3:**
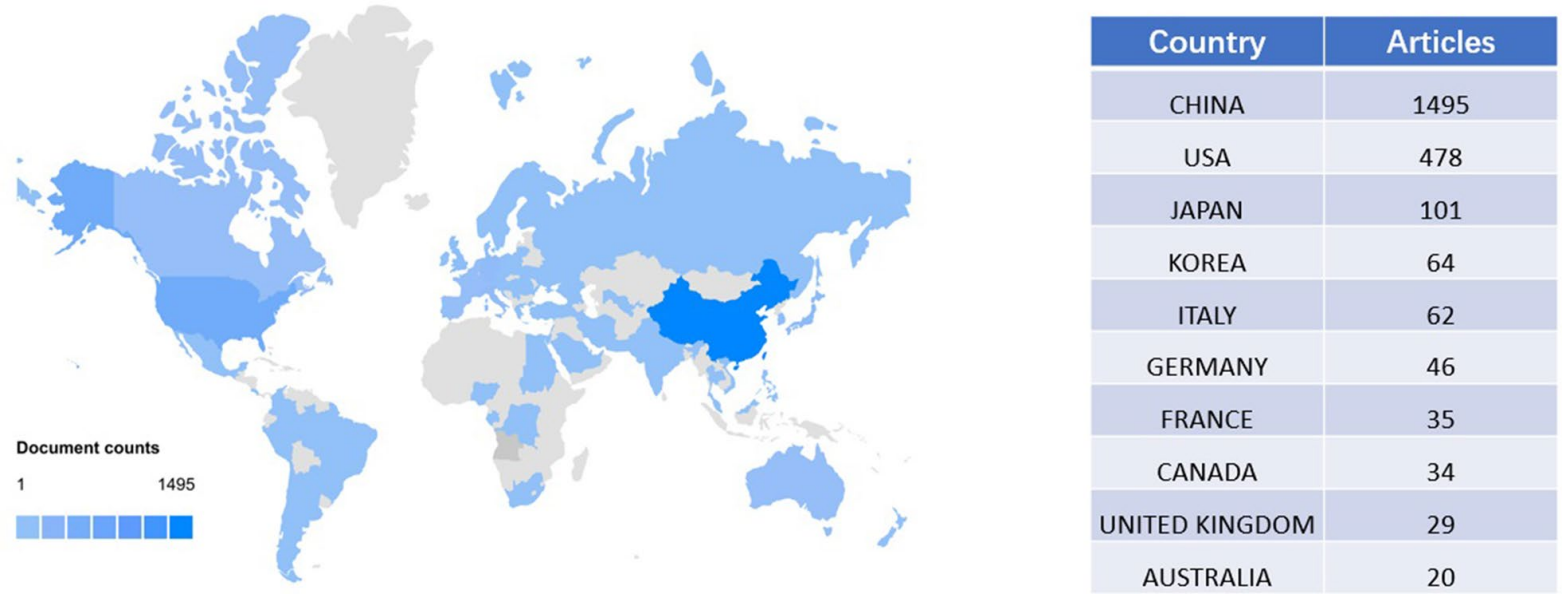
The overview of publication number of each country

A total of 2545 articles were published from 44 countries. We analyzed the number of national publications and the result are presented in Fig. 3. Among all of the countries, China had the highest number of publications (*n* = 1495, 58.7%), followed by the USA (*n* = 478, 18.8%) and Japan (*n* = 101, 4.0%). The top three most productive countries contributed to more than 80% of the total publications and the remaining countries had less than 100. To clarify the collaborative relationships among these countries, we constructed a chord diagram in Additional file 1: Fig. S1 to visualize the cooperation**.** Most of the research collaborations were between the USA, East Asian countries, and Europe. The most frequent collaborations were between the USA and China (frequency = 126), followed by the collaborations between the USA and Japan (frequency = 55), then between the USA and the UK (frequency = 28).

**Fig. 4:**
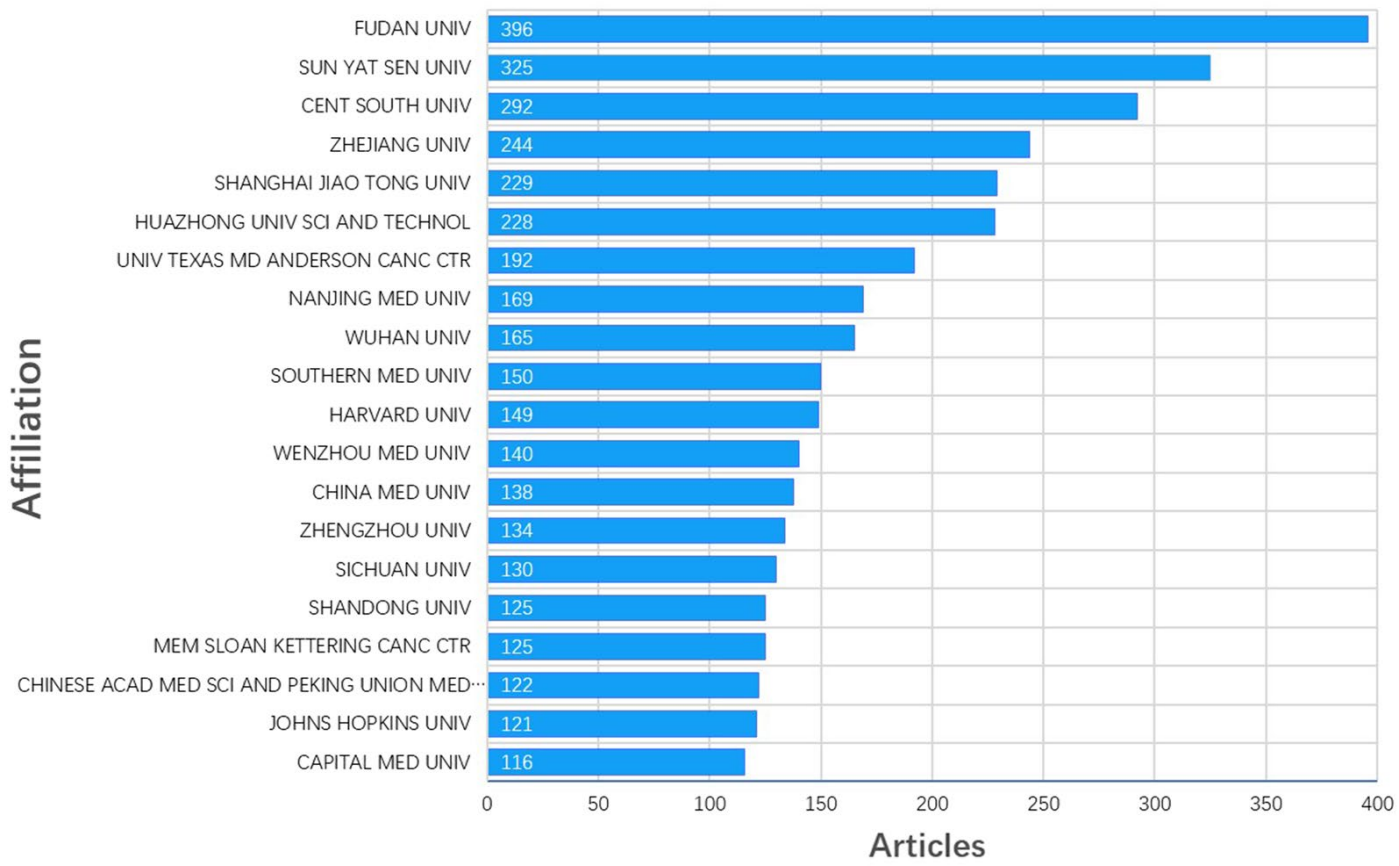
The top 20 most productive institutions in the field of TIME

We then evaluated the number of institutional publications to investigate how institutions contribute to the TIME-related research. A total of 2707 institutions contributed to the research of TIME and we the top 20 institutions are summarized in Fig. 4. Among the most productive institutions, there were 16 from China and 4 from the USA. Fudan University ranked first with 396 publications. Inter-institutional collaborations were further investigated using clustering network analysis with co-authorship analysis and time-overlapping network analysis. In the network analysis, the size of the plots represented publication numbers and the same color represented a similar intensity of collaboration. In Additional file 1: Fig. S2A, we have included the top 30 institutions with the most publication numbers and formed five clusters. The cluster with the most institutions is shown in red; there are 10 in this cluster, all of which were Chinese institutions, while cluster 2 colored green contains all the 7 institutions other than those in China of the 30 included. We noticed that collaborations within countries were far more frequent than inter-country collaborations. In Additional file 1: Fig. S2B, following the lead of American hospitals, notably Massachusetts General Hospital, as pioneers in the field of TIME, researchers in Japan and China subsequently conducted numerous studies, contributing significantly to the body of research in this area.

### Analysis of authors and academic journals

More than 10,000 researchers participated in the research and publication work of TIME and the top ten authors with the most publications were all Chinese. As shown in Table 1, Wang was the most productive author who published 158 articles with an h_index of 19, followed by Zhang who published 119 articles with an h_index of 16, and Li who published 107 articles with an h_index of 16.

**Table 1:** Top ten authors with the most publications in the field of TIME research

Rank	Author	Publications	h_index
1	Wang Y	158	19
2	Zhang Y	119	16
3	Li Y	107	16
4	Wang X	105	18
5	Zhang J	95	14
6	Wang J	87	13
7	Wang Z	87	14
8	Li J	84	16
9	Liu Y	82	11
10	Zhang X	78	14

To further investigate the collaborative relationships among researchers, we used clustering network analysis to illustrate and visualize these results. As shown in Additional file 1: Fig. S3A, 109 authors with publication numbers no less than 7 were integrated into 17 clusters. Chinese and Japanese researchers showed more collaborative tendencies than other countries, which suggested that collaborations among international researchers in the field of TIME should be further enhanced. In the time-overlapping network analysis, as shown in Additional file 1: Fig. S3B, we have observed emerging research network trends in China compared to researchers from other countries, indicating the growing significance of future research conducted in China. This highlighted the need to pay attention to relevant studies originating from China in the coming years.

In the analysis of journal publication patterns, we identified a total of 561 academic journals that have published articles related to TIME. We then recognized ten score sources journals according to Bradford’s law [34] (Additional file 1: Fig. S4), with Frontiers in Oncology (*n* = 178, IF 2021 = 5.738) ranking first, followed by Frontiers in Immunology (*n* = 169, IF 2021 = 8.786), and Cancers (*n* = 112, IF 2021 = 6.575) (see Table 2). Among the top ten journals, 40% (4/10) were from the USA and 40% (4/10) were from Switzerland, followed by 20% (2/10) from the UK. According to journal citation reports (JCR), Q1 was assigned to 50% (5/10) of the journals and Q2 to 40% (4/10).

**Table 2:** Top ten journals with the most publications in the field of TIME research

Rank	Journals	Articles	Country	IF	JCR-c
1	Frontiers in Oncology	178	Switzerland	5.738	Q2
2	Frontiers in Immunology	169	Switzerland	8.786	Q1
3	Cancers	112	Switzerland	6.575	Q2
4	Frontiers in Genetics	87	USA	4.772	Q2
5	Frontiers in Cell and Developmental Biology	83	Switzerland	6.081	Q3
6	Journal for ImmunoTherapy of Cancer	72	USA	12.469	Q1
7	Scientific Reports	42	UK	4.996	Q1
8	BMC Cancer	39	UK	4.638	Q1
9	Aging-US	36	USA	5.955	Q2
10	Clinical Cancer Research	33	USA	13.801	Q1

### Research hot spot analysis

#### Most cited publications

We retrospectively recognized the ten most highly cited articles, as shown in Additional file 1: Table S1, among the 2545 articles included. All of these ten articles were open access and have reached more than 400 citations, 60% (6/10) of which have reached more than 500 citations. Of these articles, one review entitled “Understanding the tumor immune microenvironment (TIME) for effective therapy”, published in 2018, is the most cited article in the field of TIME with 1798 citations [2], which was also the only review type of article. Other most cited articles focused more on basic medicine or pre-clinical medicine research [35–40], while only two articles specifically focused on exploring the TIME landscape of specific cancer types in clinical scenarios [41, 42].

#### Citation burst analysis

In Additional file 1: Fig. S5, we searched the top 25 articles with the highest citation burst. We set the minimum duration of the burst as 1 year. The burst duration time is shown by the red line, while the time period from 2006 to September 14, 2022 is shown by the blue line. Of these articles, the article entitled “Safety, activity, and immune correlates of anti-PD-1 antibody in cancer”, published in 2012, had the most robust citation burst value (citation burst = 23.54) during 2013–2020 [43]. Additionally, the citation burst for three articles is still running strong, such as “International validation of the consensus Immunoscore for the classification of colon cancer: a prognostic and accuracy study” [44], which helped to establish a robust scoring system of immune microenvironment based on immunohistochemistry in the future treatment of colon cancer. The other two articles focused on the genomic levels and pan-cancer analysis [45, 46], which could be helpful in finding new potential frontiers in the research of TIME.

#### Keyword analysis

**Fig. 5:**
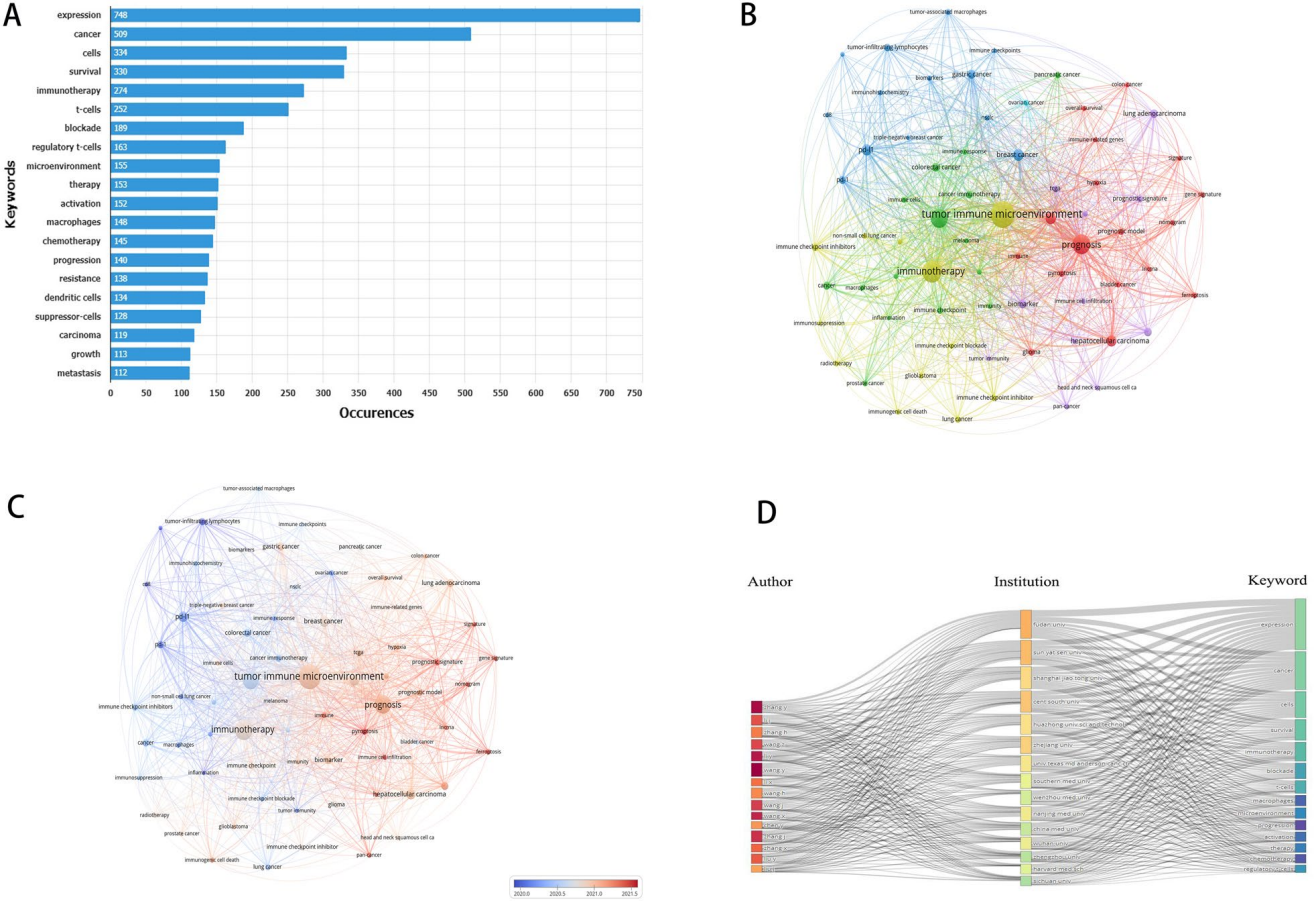
**A** The top 20 most frequent used keywords in the field of TIME; **B** clustering network analysis of keyword co-occurrence; **C** time-overlapping network analysis of keyword co-occurrence; **D** three-field plot of the keywords analysis on TIME

Among the 2545 articles we included, we recognized a total of 4062 keywords. Setting an admission criteria number of at least 20, 69 keywords were included in the study. In Fig. 5A, we listed the top 20 most frequently used keywords, where “expression” was the most relevant keyword with 748 occurrences, followed by “cancer” (*N* = 509) and “cells” (*N* = 334). No specific cancer type was listed in the most frequent keywords, whereas basic medical-related researches that focused more on the cellular level were still trending.

We then performed clustering network analysis and time-overlapping network analysis to explore the relationship among these keywords further. The size of nodes denoted the occurrence frequency of keywords, and the space distance between nodes represented the closeness of their relevance. In Fig. 5B, 69 selected keywords were separated into six clusters. In cluster 1 with the most keywords colored red, new concept such as “pyroptosis,” “ferroptosis,” “gene signature,” and “lnc-RNA” were explored in several tumor types and relevant research focused more on “prognosis” and “survival.” Cluster 2, colored green, focused on immune-related topics such as “immune cells,” “immune checkpoint,” “immune response,” and “cancer immunotherapy” in several tumor types. Cluster 3 was in blue and concentrated on widely studied immune checkpoints represented by “pd-1” and “pd-l1”. In other clusters, several cancer types with immune-related topics and microenvironment components intertwined with little regularity. In the time-overlapping network analysis shown in Fig. 5C, where the node colors are determined by the average time of each keyword’s occurrence year, we noticed earlier research focused more on “immune checkpoint inhibitors,” “pd-1,” and “pd-l1.” As research progressed, recent studies focused more on the topics such as “gene signature,” “ferroptosis,” “pan cancer,” and “prognostic signatures.”

To better understand the relationships between the authors, relevant institutions, and keywords, the three-field plot in Fig. 5D represents the connections by gray links, in which the shades of colors represent the frequency of publications. In Additional file 1: Fig. S6, we listed the top 25 keywords with the most citation bursts from 2006 to September 14, 2022. The keywords “pd1 blockade” (2017–2020) (citation burst = 14.55), “checkpoint blockade” (2015–2019) (citation burst = 11.56), and “tumor associated macrophage” (2017–2020) (citation burst = 10.98) have received relatively more attention over time. Keywords such as “tumor infiltrating lymphocyte” (2018–2020) and “immune cell” (2019–2020) have been used more recently. However, in the recent 2 years, there is still a lack of enough hot research trend burst, indicating that popular research topics in the future remain to be explored.

## Discussion

In this research, we implemented a comprehensive bibliometric analysis from the Web of Science database incorporating 2545 articles from 2006 to September 14, 2022, in the research field of TIME. These articles were published in 561 journals from 2707 institutions in 44 countries. We observed that articles in the field of TIME had shown a growing trend over the last decades, indicating that this field has achieved sufficient attention in recent years. As James and Tasuku Honjo were awarded the Nobel Prize in 2018 for their research on cancer immunotherapy using immune checkpoint inhibitors, we found publications in the field of TIME reached a critical turning point in 2019 and have been growing significantly until today. The keyword “Immunotherapy,” which emerged as one of the most frequently occurring keywords in our study, signified that research on the TIME has predominantly focused on clinical value-oriented investigations. Furthermore, the clinical significance of immunotherapy has reached new heights in recent years.

In our bibliometric study, we noticed most of the articles were published by countries from East Asia (China, Japan, Korea), North America (the USA, Canada), and Europe (Italy, Germany, France). The top three countries in publication numbers (China, the USA, and Japan) contribute to more than 80% of TIME-related publications. Such a phenomenon verified that enough governmental economic expenditure ensures academic output capability [47]. For example, the USA had the highest annual health expenditure in 2020, with USD 10,202 per resident surpassing any other country [48] and had the second publication numbers. Among the top ten most productive countries, China was the only developing country that contributed to 58.7% of the publications with total health expenditures of 7217.5 billion RMB in 2020 [49]. Although China has reached nearly three times the publications of the USA, the citation frequency of China publications ranks second and is far behind the USA. International cooperation with the USA was also the most frequent, as the top nine inter-country collaboration rankings were all between the USA and other countries. In our research of authors and institutions, Chinese authors were found to be the most productive and similarly Chinese institutions. Above all, Chinese researchers contributed a great deal of effort and outputs to the field of TIME, despite that research impacts and international collaborations still had a long way to go.

Analysis of journals can help researchers find intriguing topics and select proper journals when submitting TIME-related manuscripts [50]. All of the top ten journal publishers with the most publications in the field of TIME are located in the USA or Europe. Frontiers in Oncology and Frontiers in Immunology were the top two most productive journals, significantly surpassing other journals in publication numbers. TIME-related research fields covered various types of tumors and cutting edge of immunotherapy which were highly compatible with the research areas of these two journals [51–54]. Impact factor (IF) refers to the average number of citations for articles published in a journal in a given year and journal citation report (JCR) is a quantitative partitioning index to range journals from the top quartile (Q1) to the last quartile (Q4) based on the journals’ impact factors and research field. Both these two indices were commonly used to evaluate the influences of journals. We found no significant associations between the number of publications, IF value, and JCR value among the journals in our study, indicating different preferred tendencies of these journals. Researchers thus have more choices in selecting proper journals to submit according to their research subtypes.

Keyword analysis could reflect the current status and hot spots of research in the field of TIME. We noticed that several aspects of this field could be discussed separately. Firstly, “PD-1” and “PD-L1” were classified as representative keywords in one dependent cluster in our clustering network analysis and showed significant status in the citation burst analysis, both of which were exemplary benchmarks for tumor-related immunotherapy. Signals through the PD-1 pathway mainly involve the process of negative regulation of T cell activation when engaging its ligand PD-L1 [55]. The lack of PD-1 expression on T cells contributes to enhanced autoimmunity in mice [56]. On the contrary, high expression of PD-1 and PD-L1, commonly found in tumor immune microenvironment, can induce immune escape [57]. Anti-PD-1 or PDL-1 immunotherapies have succeeded in several clinical trials and have been implemented into clinical scenarios for various cancer types [58–61]. However, many patients have not got satisfactory remission after PD-1 therapy. Researchers never stop searching for effective biomarkers to predict the clinical efficacy of PD-1 or PD-L1-related immunotherapy and select proper patients who will benefit from the treatment. As the most widely selected predictor, tumor PD-L1 expression, which correlates with infiltrating immune cells, can reflect anti-PD-1 therapy response [62]. Increased positive predictive values of agent efficacy were observed with PD-L1 expression cutoff value growth in urothelial cancer [63], non-small cell lung cancer (NSCLC) [64], and melanoma [65] treated with pembrolizumab. Apart from exploring immune checkpoints as tumor biomarkers for predicting disease prognosis, researchers also explored combination therapy based on anti-PD-1 antibodies, such as anti-angiogenesis targeting therapy [66, 67] and CDK4/6 inhibitor [68] which could help improve clinical outcomes. Above all, we can clearly observe the significance of PD-1-related immunotherapy and its vast research prospects. Whether exploring it at the cellular level or from the perspective of clinical benefits, research on immune checkpoints and related treatments is expected to remain highly active in the future. The importance of PD-1 and its therapeutic implications have already made a significant impact in the field of cancer immunotherapy. Consequently, the research interest in this area is expected to persist and thrive as scientists continue to explore novel strategies, combination therapies, and biomarkers to further enhance treatment outcomes and expand the applications of PD-1-related immunotherapy.

As diverse types of TIME were observed in different tumors, pan-cancer analysis and clinical trials of immunotherapy could bring remarkable benefits to patients. In the research of specific tumor treatment, the analysis of keywords in our study revealed notable attention from researchers toward several tumor types. “Breast cancer,” “hepatocellular carcinoma,” “lung adenocarcinoma,” and “colorectal cancer” were the most mentioned cancer-type keywords relatively. While some of these tumors may be more common, it is important to attribute the extensive research conducted in the field of immunotherapy across these cancer types. From a clinical perspective, immunotherapy has been successful in several cancer types but still fails to get satisfactory therapeutic effects in others. Thus, exploring the composition and mechanism of TIME becomes crucial in enhancing our understanding of immunotherapy across various cancer types.

Different cancer types exhibit distinct research focuses based on their tumor characteristics. In the immunotherapy of advanced NSCLC, PD-L1 expression served as an essential measurement to evaluate feasible treatment options. Pembrolizumab was recommended as the first-line therapy for advanced or metastatic NSCLC without sensitizing EGFR or ALK alterations and with low PD-L1 expression [69]. For PD-L1 expression over 50%, atezolizumab [70] and cemiplimab-rwlc [71] were also added to the first-line recommendation. In addition to immunotherapy, targeted therapies based on EGFR mutation provided many options for patients with advanced NSCLC [72–74]. Based on the current treatment background, researchers focused on the relationships between EGFR-related treatment and TIME features. One study demonstrated that EGFR-TKI treatment was associated with changes in the TIME in CD8+ TIL density and PD-L1 expression level [75], and that EGFR-TKI could down-regulate PD-L1 in EGFR mutant NSCLC was confirmed in one previous study [76]. With the wide implementation of single cell sequencing in recent years, EGFR mutant lung adenocarcinoma was revealed with suppressive TIME [77]. These studies offered valuable insights into the treatment effectiveness of NSCLC and patient classification, highlighting the need for further research to optimize immunotherapy strategies based on different types of TIME.

Similar research mode could be seen from different tumor types according to heterogenous TIME features, thus bringing different research focus. In the research field of glioma, restricted by drug delivery routes via the blood–brain barrier (BBB) and the immunosuppressive tumor microenvironment, traditional chemotherapy is severely hampered. Various nanoparticles which could penetrate BBB were designed to induce apoptosis of tumor cells, antiangiogenesis, and tumor immune microenvironment regulation, which provided a promising avenue for viable treatment [78, 79]. Moreover, triple-negative breast cancer (TNBC) lacks targeted therapy owing to the receptor expression profile and genotype. Clinical efforts of immunotherapy have been made to convert nonresponders to responders, strengthen effective responses, and reduce resistance to immunotherapy [80]. Thus, TIME subtypes of TNBC were defined according to CD8+ T cell infiltration, PD-L1 expression, and enrichment signatures of specific functions to predict prognosis and identify potential therapeutic targets [81]. Focusing on the TIME features of TNBC, a structured TIME was elucidated using the method of multiplexed ion beam imaging by time of flight (MIBI-TOF) to select proper drugs and increase therapeutic response [82]. Overall, these research approaches highlighted the significance of understanding the specific TIME features in different tumor types to develop effective treatment strategies. In the near future, there is a growing need for extensive clinical research and implementation of precise immunotherapy tailored to different types of cancer.

To achieve precise immunotherapy, the frontier of clinical research also requires a new landscape and breakthroughs in basic medical research, as researchers have confirmed that immune cell infiltration status with heterogeneous cell types could predict tumor prognosis and guide treatment [83]. As mentioned in “Introduction”, a TIME model was established according to TIL status. CD8^+^ TIL plays a vital role in killing tumor cells directly and keeping immune surveillance, and higher CD8^+^ TIL density was found with prolonged overall survival (OS) in patients with HPV+ oropharyngeal squamous cell carcinoma [84]. TIL status information was mainly obtained from pathologic samples. Thus, Saltz and his colleagues developed a deep learning method on pathology images to elaborate the relationship between the spatial organization of TIL and molecular correlation [85]. In addition, great efforts were also made to change tumor-infiltrating immune cell type, such as changing tumor-associated macrophages (TAMs) from M2 to tumor-killing M1 phenotype [39, 86] and to find feasible approaches to rebuild TIME from approaches such as vascular and lymphatic vessel normalization [87, 88]. Other studies including those focusing on the function of intestinal flora in shaping TIME [35] and how to regulate the pyroptosis process in TIME [89] provided different angles in developing new treatment strategies. In recent years, nanomedicine has been deemed to have great potential to improve the landscape of cancer immunotherapy [90, 91]. Moreover, new strategies such as organoid modeling, macrophage engineering, and deep learning in imaging have provided various future directions in immunotherapy [37, 85, 92]. By embracing these interdisciplinary approaches and continuous research efforts in the immunocyte pattern, we can pave the way for personalized and targeted immunotherapy that will benefit a wide range of cancer patients in the near future.

To our knowledge, this study is the first systematic analysis of TIME-related publications which could provide an informative guide for researchers working in or focusing on this field. Inevitably, several limitations should be admitted. Firstly, we only analyzed pieces of literature from WOSCC alone, excluding data from other widely searched databases such as PubMed, Embase, and articles published in other languages. Secondly, as TIME was a broad concept and included different research directions, studies that concentrated on one subfield of TIME might refuse to select TIME as a keyword. This could lead to the omission of proper articles. Thirdly, the WoSCC database contained different levels of databases, such as ESCI provided earlier visibility for sources under evaluation. It is worth noting that the inclusion of these databases may have potential implications for the precision and generalizability of our study. Finally, it is worth noting that the publication trend in the field of TIME has shown a significant increase in the number of articles in recent years. However, it is important to acknowledge that many of these recently published articles may not have received the attention they deserve, possibly due to their low citation frequency.

## Conclusion

Generally, research in the field of TIME has witnessed significant growth in terms of annual publications. China emerged as the leading country in terms of productivity, with numerous productive authors and institutions contributing to the field. On the other hand, the USA demonstrated the highest citation frequency, indicating the impact of research from this country. Among the journals publishing articles in this field, Frontiers in Oncology and Frontiers in Immunology stood out as the most productive ones. The prevailing research direction in this field has been predominantly clinical oriented, with a focus on exploring immunotherapy in various cancer types. Our researchers offered a thorough understanding of TIME and summarized these areas: “immune checkpoint-based immunotherapy”, “precise immunotherapy”, and “immunocyte pattern” as frontiers and focal points in the upcoming years which may provide valuable opportunities for further investigation.

## Supplementary Information

**Additional file 1: Figure S1.** International collaboration and high frequency of collaboration countries in the field of TIME research. **Figure S2.** Clustering network analysis and time-overlapping network analysis for institutional co-authorship. **Figure S3.** Clustering network analysis and time-overlapping network analysis for co-authorship. **Figure S4.** Ten core sources academic journals according to the Bradford’s Law. **Figure S5.** Top 25 cited references with the strongest citation bursts in the field of TIME research. **Figure S6.** Top 25 keywords with the strongest citation bursts in the field of TIME research. **Table S1.** The top 10 cited publications.

## Data Availability

The original contributions presented in the study are included in the article/Additional file. Further inquiries can be directed to the corresponding author.

## References

[1] Hanahan D (2022). Hallmarks of cancer: new dimensions. Cancer Discov.

[2] Binnewies M, Roberts EW, Kersten K, Chan V, Fearon DF, Merad M, Coussens LM (2018). Understanding the tumor immune microenvironment (TIME) for effective therapy. Nat Med.

[3] Evans RA, Diamond MS, Rech AJ, Chao T, Richardson MW, Lin JH, Bajor DL (2016). Lack of immunoediting in murine pancreatic cancer reversed with neoantigen. JCI Insight.

[4] Mlecnik B, Bindea G, Angell HK, Maby P, Angelova M, Tougeron D, Church SE (2016). Integrative analyses of colorectal cancer show immunoscore is a stronger predictor of patient survival than microsatellite instability. Immunity.

[5] Li J, Yuan S, Norgard RJ, Yan F, Sun YH, Kim IK, Merrell AJ (2021). Epigenetic and transcriptional control of the epidermal growth factor receptor regulates the tumor immune microenvironment in pancreatic cancer. Cancer Discov.

[6] Chen Y, Zheng X, Wu C (2021). The role of the tumor microenvironment and treatment strategies in colorectal cancer. Front Immunol.

[7] Dieu-Nosjean MC, Giraldo NA, Kaplon H, Germain C, Fridman WH, Sautes-Fridman C (2016). Tertiary lymphoid structures, drivers of the anti-tumor responses in human cancers. Immunol Rev.

[8] Sumimoto H, Imabayashi F, Iwata T, Kawakami Y (2006). The BRAF-MAPK signaling pathway is essential for cancer-immune evasion in human melanoma cells. J Exp Med.

[9] Khalili JS, Liu S, Rodriguez-Cruz TG, Whittington M, Wardell S, Liu C, Zhang M (2012). Oncogenic BRAF(V600E) promotes stromal cell-mediated immunosuppression via induction of interleukin-1 in melanoma. Clin Cancer Res.

[10] Fassoulaki A, Tsaroucha A, Micha G, Soulioti E (2021). Bibliometric analysis of alternative performance metrics for peri-operative, critical care and pain medicine journals. Anaesthesia.

[11] Ho WJ, Danilova L, Lim SJ, Verma R, Xavier S, Leatherman JM, Sztein MB (2020). Viral status, immune microenvironment and immunological response to checkpoint inhibitors in hepatocellular carcinoma. J Immunother Cancer.

[12] Ding Z, Dong Z, Chen Z, Hong J, Yan L, Li H, Yao S (2021). Viral status and efficacy of immunotherapy in hepatocellular carcinoma: a systematic review with meta-analysis. Front Immunol.

[13] Wang S, Zhou H, Zheng L, Zhu W, Zhu L, Feng D, Wei J (2021). Global trends in research of macrophages associated with acute lung injury over past 10 years: a bibliometric analysis. Front Immunol.

[14] de CastilhosGhisi N, Zuanazzi NR, Fabrin TMC, Oliveira EC (2020). Glyphosate and its toxicology: a scientometric review. Sci Total Environ.

[15] Wu K, Liu Y, Liu L, Peng Y, Pang H, Sun X, Xia D (2022). Emerging trends and research foci in tumor microenvironment of pancreatic cancer: a bibliometric and visualized study. Front Oncol.

[16] Chen P, Du Z, Wang J, Liu Y, Zhang J, Liu D (2021). A bibliometric analysis of the research on hematological tumor microenvironment. Ann Transl Med.

[17] Zhang Y, Huo L, Wei Z, Tang Q, Sui H (2022). Hotspots and frontiers in inflammatory tumor microenvironment research: a scientometric and visualization analysis. Front Pharmacol.

[18] Shen H, Wang L, Zhang Y, Huang G, Liu B (2023). Knowledge mapping of image-guided tumor ablation and immunity: a bibliometric analysis. Front Immunol.

[19] Zhou F, Liu Y, Liu C, Wang F, Peng J, Xie Y, Zhou X (2023). Knowledge landscape of tumor-associated macrophage research: a bibliometric and visual analysis. Front Immunol.

[20] Meho LI, Yang K (2007). Impact of data sources on citation counts and rankings of LIS faculty: web of science versus scopus and google scholar. J Am Soc Inform Sci Technol.

[21] You Y, Li W, Liu J, Li X, Fu Y, Ma X (2021). Bibliometric review to explore emerging high-intensity interval training in health promotion: a new century picture. Front Public Health.

[22] You Y, Wang D, Liu J, Chen Y, Ma X, Li W (2022). Physical exercise in the context of air pollution: an emerging research topic. Front Physiol.

[23] You Y, Wang D, Wang Y, Li Z, Ma X (2021). A bird’s-eye view of exercise intervention in treating depression among teenagers in the last 20 years: a bibliometric study and visualization analysis. Front Psychiatry.

[24] Jiang ST, Liu YG, Zhang L, Sang XT, Xu YY, Lu X (2022). Immune-related adverse events: a bibliometric analysis. Front Immunol.

[25] Sood SK, Rawat KS, Kumar D (2022). Analytical mapping of information and communication technology in emerging infectious diseases using CiteSpace. Telemat Inform.

[26] Sood SK, Rawat KS, Kumar D (2022). A visual review of artificial intelligence and Industry 4.0 in healthcare. Comput Electr Eng.

[27] van den Hoven AF, Keijsers RGM, Lam M, Glaudemans A, Verburg FA, Vogel WV, Lavalaye J (2023). Current research topics in FAPI theranostics: a bibliometric analysis. Eur J Nucl Med Mol Imaging.

[28] Chadegani AA, Salehi H, Yunus MM, Farhadi H, Fooladi M, Farhadi M, Ebrahim NA (2013). A comparison between two main academic literature collections: web of science and scopus databases. Asian Soc Sci.

[29] Team RC. R: a language and environment for statistical computing. R Foundation for Statistical Computing; 2013. https://www.R-project.org/.

[30] van Eck NJ, Waltman L (2010). Software survey: VOSviewer, a computer program for bibliometric mapping. Scientometrics.

[31] Chen C (2006). CiteSpace II: detecting and visualizing emerging trends and transient patterns in scientific literature. J Am Soc Inf Sci Technol.

[32] van Eck NJ, Waltman L (2017). Citation-based clustering of publications using CitNetExplorer and VOSviewer. Scientometrics.

[33] van Eck NJ, Waltman L, van Raan AF, Klautz RJ, Peul WC (2013). Citation analysis may severely underestimate the impact of clinical research as compared to basic research. PLoS ONE.

[34] Venable GT, Shepherd BA, Loftis CM, McClatchy SG, Roberts ML, Fillinger ME, Tansey JB (2016). Bradford’s law: identification of the core journals for neurosurgery and its subspecialties. J Neurosurg.

[35] Kostic AD, Chun E, Robertson L, Glickman JN, Gallini CA, Michaud M, Clancy TE (2013). *Fusobacterium nucleatum* potentiates intestinal tumorigenesis and modulates the tumor-immune microenvironment. Cell Host Microbe.

[36] Deng L, Liang H, Burnette B, Beckett M, Darga T, Weichselbaum RR, Fu YX (2014). Irradiation and anti-PD-L1 treatment synergistically promote antitumor immunity in mice. J Clin Invest.

[37] Neal JT, Li X, Zhu J, Giangarra V, Grzeskowiak CL, Ju J, Liu IH (2018). Organoid modeling of the tumor immune microenvironment. Cell.

[38] De Henau O, Rausch M, Winkler D, Campesato LF, Liu C, Cymerman DH, Budhu S (2016). Overcoming resistance to checkpoint blockade therapy by targeting PI3Kgamma in myeloid cells. Nature.

[39] Rodell CB, Arlauckas SP, Cuccarese MF, Garris CS, Li R, Ahmed MS, Kohler RH (2018). TLR7/8-agonist-loaded nanoparticles promote the polarization of tumour-associated macrophages to enhance cancer immunotherapy. Nat Biomed Eng.

[40] Koyama S, Akbay EA, Li YY, Herter-Sprie GS, Buczkowski KA, Richards WG, Gandhi L (2016). Adaptive resistance to therapeutic PD-1 blockade is associated with upregulation of alternative immune checkpoints. Nat Commun.

[41] DeNardo DG, Brennan DJ, Rexhepaj E, Ruffell B, Shiao SL, Madden SF, Gallagher WM (2011). Leukocyte complexity predicts breast cancer survival and functionally regulates response to chemotherapy. Cancer Discov.

[42] Senbabaoglu Y, Gejman RS, Winer AG, Liu M, Van Allen EM, de Velasco G, Miao D (2016). Tumor immune microenvironment characterization in clear cell renal cell carcinoma identifies prognostic and immunotherapeutically relevant messenger RNA signatures. Genome Biol.

[43] Topalian SL, Hodi FS, Brahmer JR, Gettinger SN, Smith DC, McDermott DF, Powderly JD (2012). Safety, activity, and immune correlates of anti-PD-1 antibody in cancer. N Engl J Med.

[44] Pages F, Mlecnik B, Marliot F, Bindea G, Ou FS, Bifulco C, Lugli A (2018). International validation of the consensus Immunoscore for the classification of colon cancer: a prognostic and accuracy study. Lancet.

[45] Gao J, Aksoy BA, Dogrusoz U, Dresdner G, Gross B, Sumer SO, Sun Y (2013). Integrative analysis of complex cancer genomics and clinical profiles using the cBioPortal. Sci Signal.

[46] Gentles AJ, Newman AM, Liu CL, Bratman SV, Feng W, Kim D, Nair VS (2015). The prognostic landscape of genes and infiltrating immune cells across human cancers. Nat Med.

[47] Lerman TT, Reitblat O, Reitblat T (2021). Scientific productivity in rheumatology among countries of the organisation for economic co-operation and development and its correlation to national economic indicators. J Clin Rheumatol.

[48] Sun HL, Bai W, Li XH, Huang H, Cui XL, Cheung T, Su ZH (2022). Schizophrenia and inflammation research: a bibliometric analysis. Front Immunol.

[49] National Bureau of Statistics. Health expenditures in China; 2020. https://data.stats.gov.cn/easyquery.htm?cn=C01.

[50] Shen J, Shen H, Ke L, Chen J, Dang X, Liu B, Hua Y (2022). Knowledge mapping of immunotherapy for hepatocellular carcinoma: a bibliometric study. Front Immunol.

[51] Xu Q, Chen S, Hu Y, Huang W (2021). Landscape of immune microenvironment under immune cell infiltration pattern in breast cancer. Front Immunol.

[52] Yu L, Ding Y, Wan T, Deng T, Huang H, Liu J (2021). Significance of CD47 and its association with tumor immune microenvironment heterogeneity in ovarian cancer. Front Immunol.

[53] Hiltbrunner S, Mannarino L, Kirschner MB, Opitz I, Rigutto A, Laure A, Lia M (2021). Tumor immune microenvironment and genetic alterations in mesothelioma. Front Oncol.

[54] Yang J, Hong S, Zhang X, Liu J, Wang Y, Wang Z, Gao L (2021). Tumor immune microenvironment related gene-based model to predict prognosis and response to compounds in ovarian cancer. Front Oncol.

[55] Sharpe AH, Pauken KE (2018). The diverse functions of the PD1 inhibitory pathway. Nat Rev Immunol.

[56] Nishimura H, Okazaki T, Tanaka Y, Nakatani K, Hara M, Matsumori A, Sasayama S (2001). Autoimmune dilated cardiomyopathy in PD-1 receptor-deficient mice. Science.

[57] Iwai Y, Ishida M, Tanaka Y, Okazaki T, Honjo T, Minato N (2002). Involvement of PD-L1 on tumor cells in the escape from host immune system and tumor immunotherapy by PD-L1 blockade. Proc Natl Acad Sci USA.

[58] Pardoll DM (2012). The blockade of immune checkpoints in cancer immunotherapy. Nat Rev Cancer.

[59] Brahmer JR, Tykodi SS, Chow LQ, Hwu WJ, Topalian SL, Hwu P, Drake CG (2012). Safety and activity of anti-PD-L1 antibody in patients with advanced cancer. N Engl J Med.

[60] Garon EB, Rizvi NA, Hui R, Leighl N, Balmanoukian AS, Eder JP, Patnaik A (2015). Pembrolizumab for the treatment of non-small-cell lung cancer. N Engl J Med.

[61] Finn RS, Ryoo BY, Merle P, Kudo M, Bouattour M, Lim HY, Breder V (2020). Pembrolizumab as second-line therapy in patients with advanced hepatocellular carcinoma in KEYNOTE-240: a randomized, double-blind, Phase III Trial. J Clin Oncol.

[62] Taube JM, Klein A, Brahmer JR, Xu H, Pan X, Kim JH, Chen L (2014). Association of PD-1, PD-1 ligands, and other features of the tumor immune microenvironment with response to anti-PD-1 therapy. Clin Cancer Res.

[63] Balar AV, Castellano D, O'Donnell PH, Grivas P, Vuky J, Powles T, Plimack ER (2017). First-line pembrolizumab in cisplatin-ineligible patients with locally advanced and unresectable or metastatic urothelial cancer (KEYNOTE-052): a multicentre, single-arm, phase 2 study. Lancet Oncol.

[64] Hui R, Garon EB, Goldman JW, Leighl NB, Hellmann MD, Patnaik A, Gandhi L (2017). Pembrolizumab as first-line therapy for patients with PD-L1-positive advanced non-small cell lung cancer: a phase 1 trial. Ann Oncol.

[65] Daud AI, Wolchok JD, Robert C, Hwu WJ, Weber JS, Ribas A, Hodi FS (2016). Programmed death-ligand 1 expression and response to the anti-programmed death 1 antibody pembrolizumab in melanoma. J Clin Oncol.

[66] Yi M, Jiao D, Qin S, Chu Q, Wu K, Li A (2019). Synergistic effect of immune checkpoint blockade and anti-angiogenesis in cancer treatment. Mol Cancer.

[67] Huang Y, Kim BYS, Chan CK, Hahn SM, Weissman IL, Jiang W (2018). Improving immune-vascular crosstalk for cancer immunotherapy. Nat Rev Immunol.

[68] Schaer DA, Beckmann RP, Dempsey JA, Huber L, Forest A, Amaladas N, Li Y (2018). The CDK4/6 inhibitor abemaciclib induces a T cell inflamed tumor microenvironment and enhances the efficacy of PD-L1 checkpoint blockade. Cell Rep.

[69] Mok TSK, Wu YL, Kudaba I, Kowalski DM, Cho BC, Turna HZ, Castro G (2019). Pembrolizumab versus chemotherapy for previously untreated, PD-L1-expressing, locally advanced or metastatic non-small-cell lung cancer (KEYNOTE-042): a randomised, open-label, controlled, phase 3 trial. Lancet.

[70] Herbst RS, Giaccone G, de Marinis F, Reinmuth N, Vergnenegre A, Barrios CH, Morise M (2020). Atezolizumab for first-line treatment of PD-L1-selected patients with NSCLC. N Engl J Med.

[71] Sezer A, Kilickap S, Gumus M, Bondarenko I, Ozguroglu M, Gogishvili M, Turk HM (2021). Cemiplimab monotherapy for first-line treatment of advanced non-small-cell lung cancer with PD-L1 of at least 50%: a multicentre, open-label, global, phase 3, randomised, controlled trial. Lancet.

[72] Yang JC, Wu YL, Schuler M, Sebastian M, Popat S, Yamamoto N, Zhou C (2015). Afatinib versus cisplatin-based chemotherapy for EGFR mutation-positive lung adenocarcinoma (LUX-Lung 3 and LUX-Lung 6): analysis of overall survival data from two randomised, phase 3 trials. Lancet Oncol.

[73] Rosell R, Carcereny E, Gervais R, Vergnenegre A, Massuti B, Felip E, Palmero R (2012). Erlotinib versus standard chemotherapy as first-line treatment for European patients with advanced EGFR mutation-positive non-small-cell lung cancer (EURTAC): a multicentre, open-label, randomised phase 3 trial. Lancet Oncol.

[74] Wu YL, Cheng Y, Zhou X, Lee KH, Nakagawa K, Niho S, Tsuji F (2017). Dacomitinib versus gefitinib as first-line treatment for patients with EGFR-mutation-positive non-small-cell lung cancer (ARCHER 1050): a randomised, open-label, phase 3 trial. Lancet Oncol.

[75] Isomoto K, Haratani K, Hayashi H, Shimizu S, Tomida S, Niwa T, Yokoyama T (2020). Impact of EGFR-TKI treatment on the tumor immune microenvironment in EGFR mutation-positive non-small cell lung cancer. Clin Cancer Res.

[76] Lin K, Cheng J, Yang T, Li Y, Zhu B (2015). EGFR-TKI down-regulates PD-L1 in EGFR mutant NSCLC through inhibiting NF-kappaB. Biochem Biophys Res Commun.

[77] Yang L, He YT, Dong S, Wei XW, Chen ZH, Zhang B, Chen WD (2022). Single-cell transcriptome analysis revealed a suppressive tumor immune microenvironment in EGFR mutant lung adenocarcinoma. J Immunother Cancer.

[78] Lin T, Zhao P, Jiang Y, Tang Y, Jin H, Pan Z, He H (2016). Blood–brain-barrier-penetrating albumin nanoparticles for biomimetic drug delivery via albumin-binding protein pathways for antiglioma therapy. ACS Nano.

[79] Qiao C, Yang J, Shen Q, Liu R, Li Y, Shi Y, Chen J (2018). Traceable nanoparticles with dual targeting and ROS response for RNAi-based immunochemotherapy of intracranial glioblastoma treatment. Adv Mater.

[80] Emens LA (2018). Breast cancer immunotherapy: facts and hopes. Clin Cancer Res.

[81] Gruosso T, Gigoux M, Manem VSK, Bertos N, Zuo D, Perlitch I, Saleh SMI (2019). Spatially distinct tumor immune microenvironments stratify triple-negative breast cancers. J Clin Invest.

[82] Keren L, Bosse M, Marquez D, Angoshtari R, Jain S, Varma S, Yang SR (2018). A structured tumor-immune microenvironment in triple negative breast cancer revealed by multiplexed ion beam imaging. Cell.

[83] Marabelle A, Kohrt H, Caux C, Levy R (2014). Intratumoral immunization: a new paradigm for cancer therapy. Clin Cancer Res.

[84] Solomon B, Young RJ, Bressel M, Urban D, Hendry S, Thai A, Angel C (2018). Prognostic significance of PD-L1(+) and CD8(+) immune cells in HPV(+) oropharyngeal squamous cell carcinoma. Cancer Immunol Res.

[85] Saltz J, Gupta R, Hou L, Kurc T, Singh P, Nguyen V, Samaras D (2018). Spatial organization and molecular correlation of tumor-infiltrating lymphocytes using deep learning on pathology images. Cell Rep.

[86] Chen D, Xie J, Fiskesund R, Dong W, Liang X, Lv J, Jin X (2018). Chloroquine modulates antitumor immune response by resetting tumor-associated macrophages toward M1 phenotype. Nat Commun.

[87] Lund AW, Wagner M, Fankhauser M, Steinskog ES, Broggi MA, Spranger S, Gajewski TF (2016). Lymphatic vessels regulate immune microenvironments in human and murine melanoma. J Clin Invest.

[88] Huang Y, Goel S, Duda DG, Fukumura D, Jain RK (2013). Vascular normalization as an emerging strategy to enhance cancer immunotherapy. Cancer Res.

[89] Erkes DA, Cai W, Sanchez IM, Purwin TJ, Rogers C, Field CO, Berger AC (2020). Mutant BRAF and MEK inhibitors regulate the tumor immune microenvironment via pyroptosis. Cancer Discov.

[90] Shi Y, Lammers T (2019). Combining nanomedicine and immunotherapy. Acc Chem Res.

[91] Kong M, Tang J, Qiao Q, Wu T, Qi Y, Tan S, Gao X (2017). Biodegradable hollow mesoporous silica nanoparticles for regulating tumor microenvironment and enhancing antitumor efficiency. Theranostics.

[92] Xia Y, Rao L, Yao H, Wang Z, Ning P, Chen X (2020). Engineering macrophages for cancer immunotherapy and drug delivery. Adv Mater.

