# Supplementary material for: Worldwide productivity and research trend of publications concerning tumor immune microenvironment (TIME): a bibliometric study
Source: Eur J Med Res. 2023 Jul 10;28:229. doi: 10.1186/s40001-023-01195-3 (PMC10332017; doi:10.1186/s40001-023-01195-3)
Supplement: Supplementary file 1 — Additional file 1: Figure S1. International collaboration and high frequency of collaboration countries in the field of TIME research. Figure S2. Clustering network analysis and time-overlapping network analysis for institutional co-authorship. Figure S3. Clustering network analysis and time-overlapping network analysis for co-authorship. Figure S4. Ten core sources academic journals according to the Bradford’s Law. Figure S5. Top 25 cited references with the strongest citation bursts in the field of TIME research. Figure S6. Top 25 keywords with the strongest citation bursts in the field of TIME research. Table S1. The top 10 cited publications. [file 40001_2023_1195_MOESM1_ESM.docx]

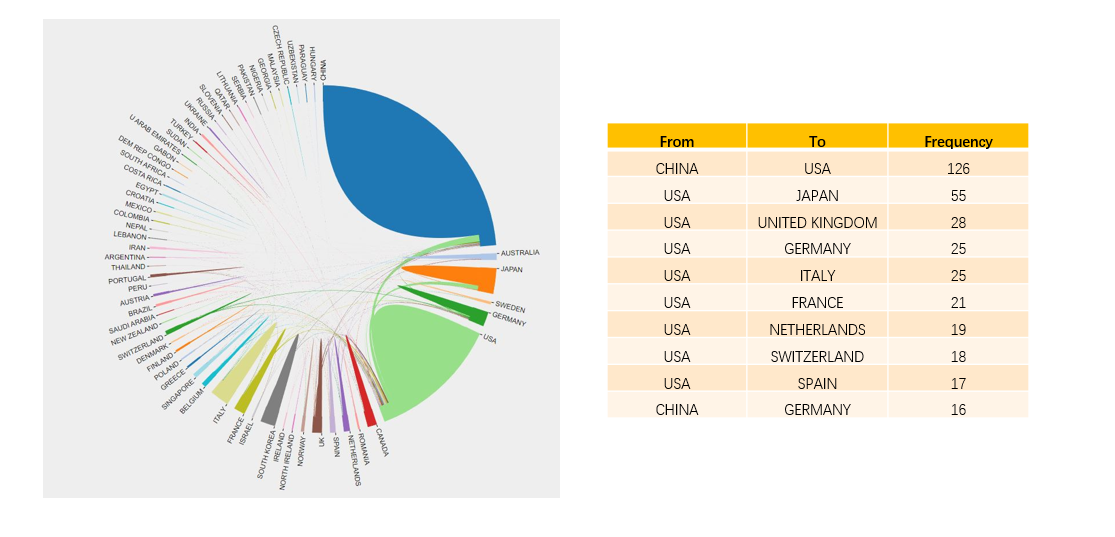


**Figure S1** International collaboration and high frequency of collaboration countries in the field of TIME research.

**A**


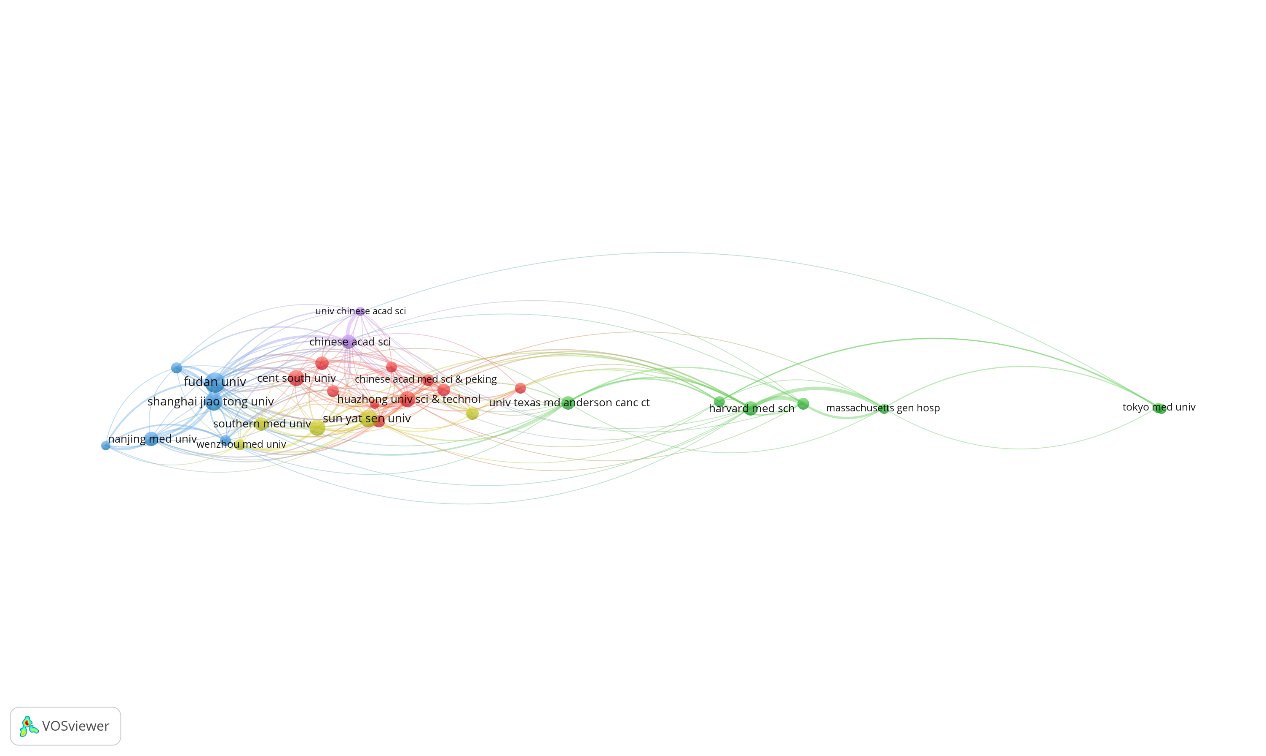


**B**


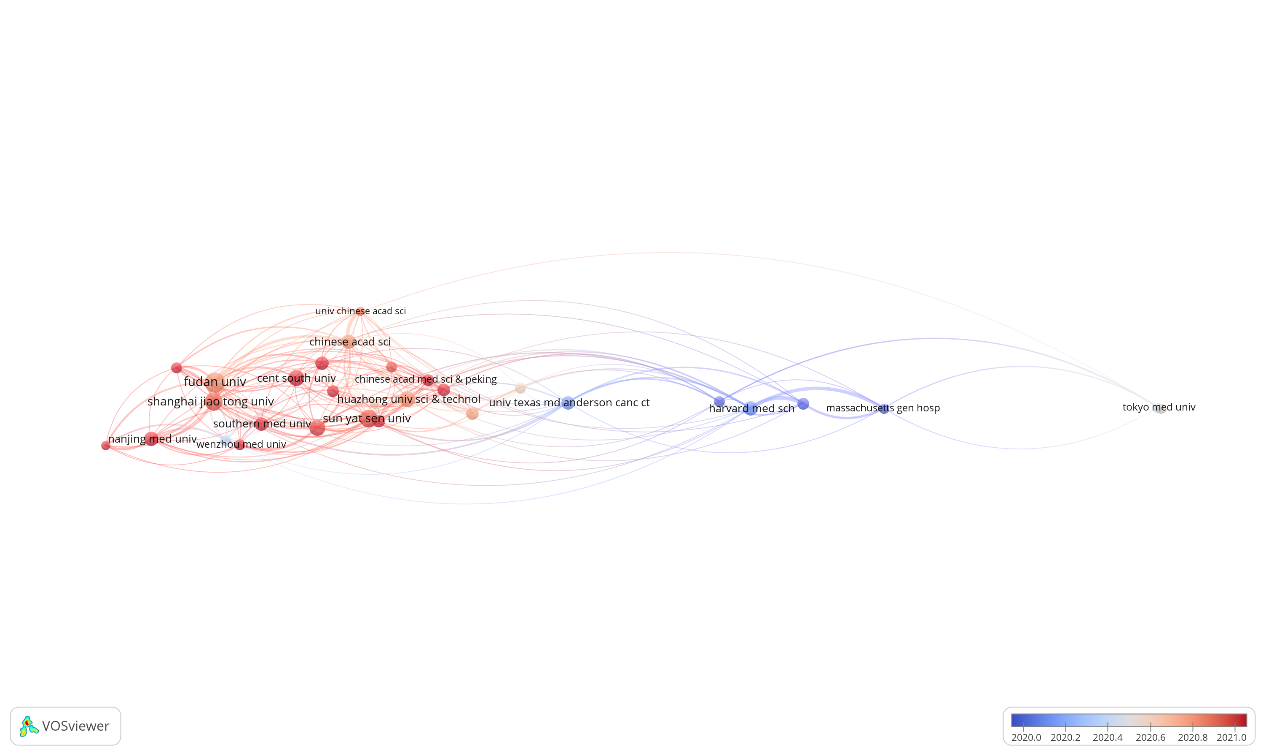


**Figure S2.** Clustering network analysis and time-overlapping network analysis for institutional co-authorship.

**A**


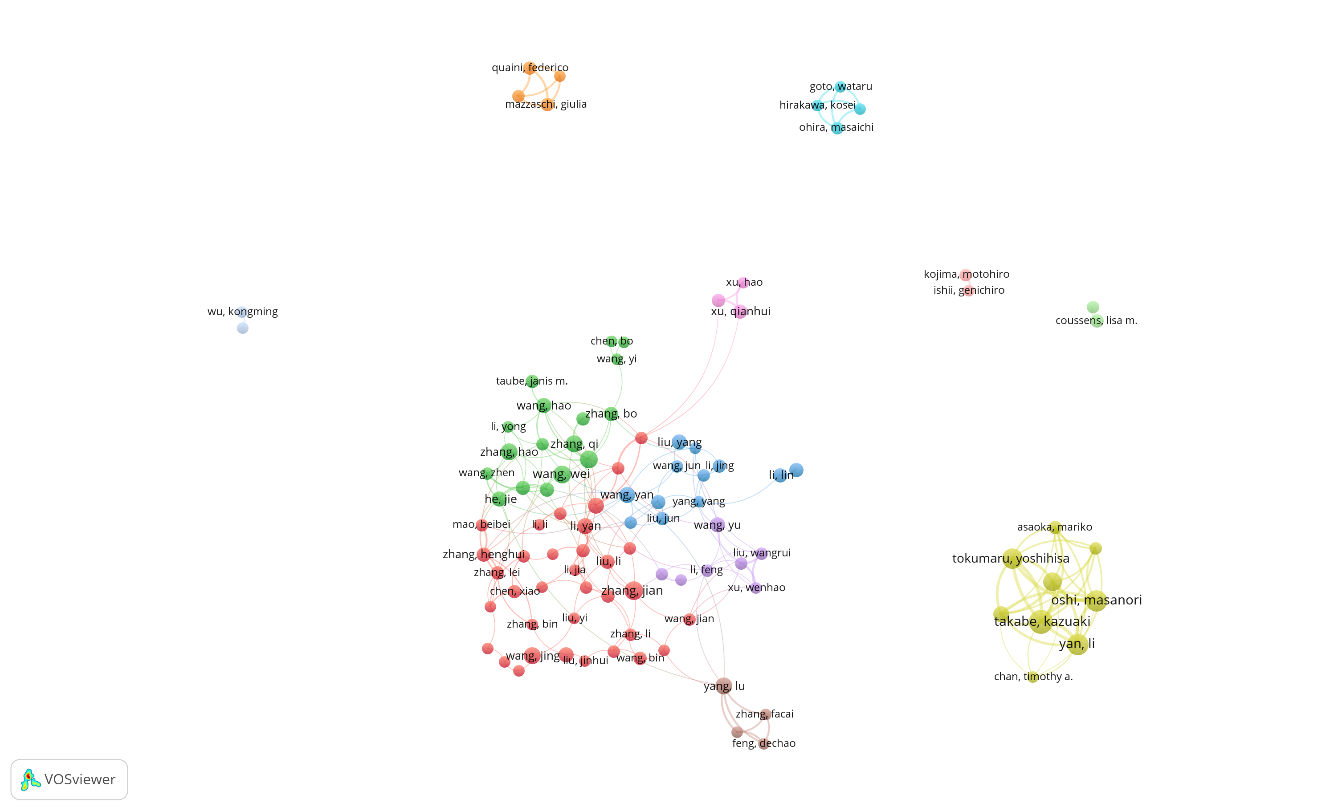


**B**
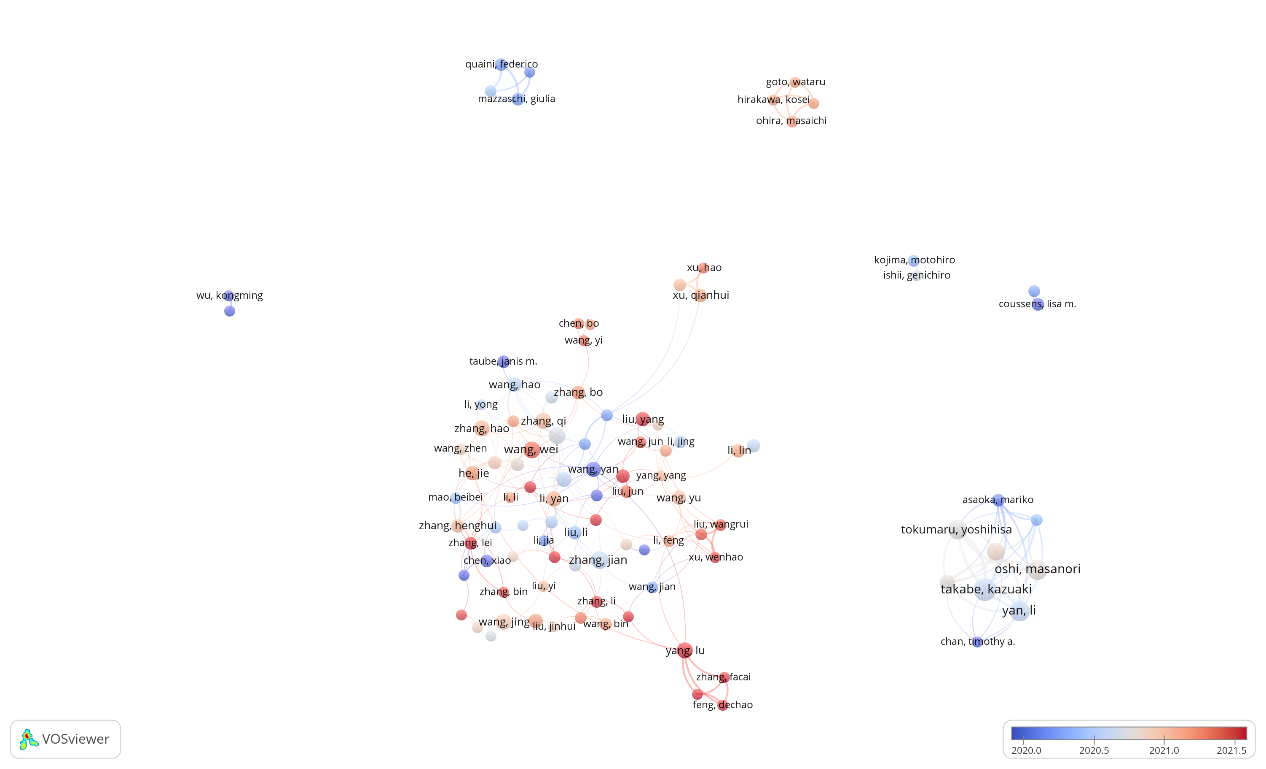


**Figure S3.** Clustering network analysis and time-overlapping network analysis for co-authorship.


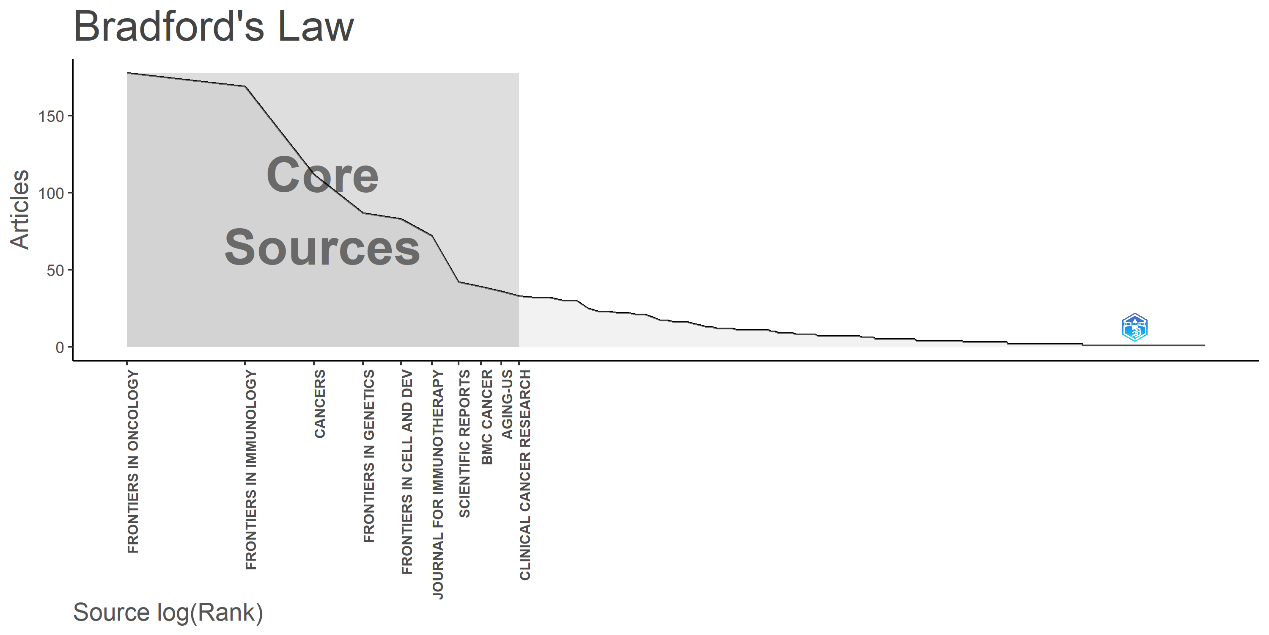


**Figure S4.** Ten core sources academic journals according to the Bradford’s Law.


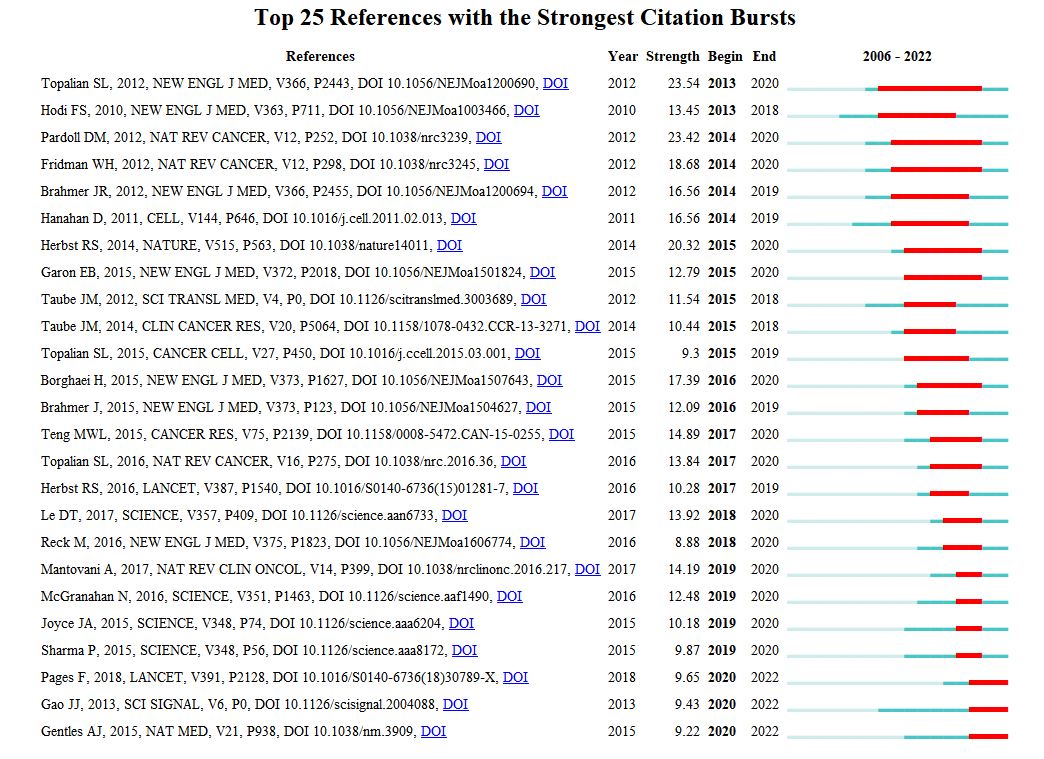


**Figure S5.** Top 25 cited references with the strongest citation bursts in the field of TIME research.


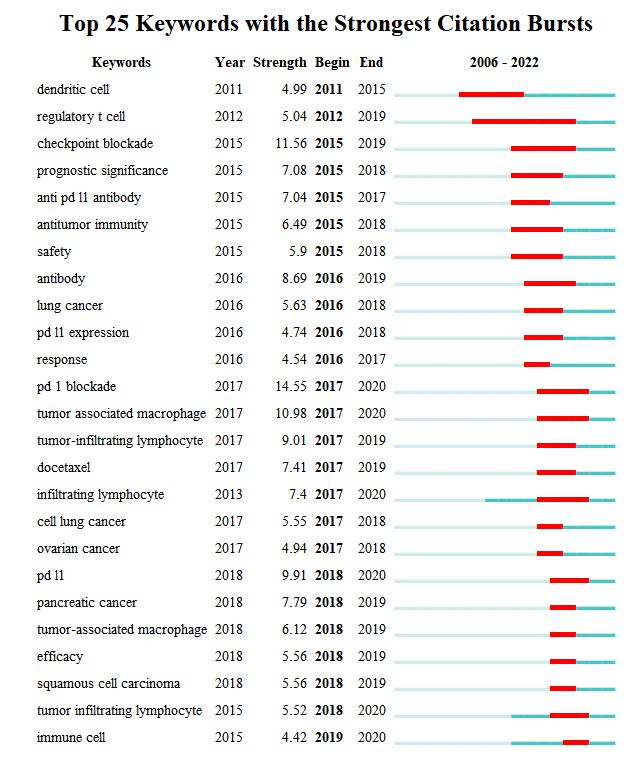


**Figure S6.** Top 25 keywords with the strongest citation bursts in the field of TIME research.

**Table S1.** The top 10 cited publications.

| Rank | Journals | Year, Journal | First author | Total citations | TC per Year |
| --- | --- | --- | --- | --- | --- |
| 1 | Understanding the tumor immune microenvironment (TIME) for effective therapy | 2018, NAT MED | BINNEWIES M | 1789 | 298.17 |
| 2 | Association of PD-1, PD-1 ligands, and other features of the tumor immune microenvironment with response to anti-PD-1 therapy | 2014, CLIN CANCER RES | TAUBE JM | 1670 | 167 |
| 3 | Fusobacterium nucleatum potentiates intestinal tumorigenesis and modulates the tumor-immune microenvironment | 2013, CELL HOST MICROBE | KOSTIC AD | 1213 | 110.27 |
| 4 | Irradiation and anti-PD-L1 treatment synergistically promote antitumor immunity in mice | 2014, J CLIN INVEST | DENG L | 1203 | 120.3 |
| 5 | Leukocyte complexity predicts breast cancer survival and functionally regulates response to chemotherapy | 2011, CANCER DISCOV | DENARDO DG | 1143 | 87.92 |
| 6 | Adaptive resistance to therapeutic PD-1 blockade is associated with upregulation of alternative immune checkpoints | 2016, NAT COMMUN | KOYAMA S | 857 | 107.13 |
| 7 | Organoid Modeling of the Tumor Immune Microenvironment | 2018, CELL | NEAL JT | 481 | 80.17 |
| 8 | Overcoming resistance to checkpoint blockade therapy by targeting PI3Kγ in myeloid cells | 2016, NATURE | DE HENAU O | 467 | 58.38 |
| 9 | TLR7/8-agonist-loaded nanoparticles promote the polarization of tumor-associated macrophages to enhance cancer immunotherapy | 2018, NAT BIOMED ENG | RODELL CB | 427 | 71.17 |
| 10 | Tumor immune microenvironment characterization in clear cell renal cell carcinoma identifies prognostic and immunotherapeutically relevant messenger RNA signatures | 2016, GENOME BIOL | SENBABAOGLU Y | 422 | 52.75 |
